# Supplementary material for: Deep learning-based detection and segmentation of diffusion abnormalities in acute ischemic stroke
Source: Commun Med (Lond). 2021 Dec 16;1:61. doi: 10.1038/s43856-021-00062-8 (PMC9053217; doi:10.1038/s43856-021-00062-8)
Supplement: Supplementary file 1 — Supplementary Information [file 43856_2021_62_MOESM1_ESM.pdf]

Deep learning-based detection and segmentation of diffusion abnormalities in acute ischemic stroke: Supplementary Information

SUPPLEMENTARY TABLES

Demographic, lesion and scanner profile	p-value:
Age in years	0.3955
Sex	0.3588
Race/Ethnicity	0.8304
NIHSS	0.7007
Symptoms onset to MRI, in hours (<6 or $\geq$6)	0.8653
Lesioned hemisphere (left, right)	0.0663
Vascular territories	0.4595
Lesion group	0.4592
Lesion volume	
Any vascular territory	0.0746
MCA	0.0581
PCA	0.7399
VB	0.3261
ACA	0.8904
Lesion contrast	
Any vascular territory	0.4986
MCA	0.7407
PCA	0.5379
VB	0.6149
ACA	0.2000
MRI voxel size (in mm^3)	0.8679
MRI magnetic field*	0.0015
MRI Manufacturer (manufacturer 1 vs 3)*	0.0004

Supplementary Table 1. Statistical testing on the demographic, lesion and image profiles distributions between training dataset (n=1390) and testing dataset (n=459). Significant differences are marked with "*".

Configuration of parameters				Dice (mean±std)	Net overlap (mean±std)
W_{fwhm}	σ_{dwi}	σ_{adc}	σ_{id}		
4	1	1	3.5	0.33±0.29	0.49±0.29
4	1	2	3.5	0.32±0.29	0.49±0.29
4	2	1	3.5	0.37±0.29	0.46±0.29
4	2	2	3.5	0.36±0.29	0.46±0.29

Supplementary Table 2. Dice and Net Overlap of the top four parameters′ configurations of the t-score method over testing dataset (n=459).

Dataset	Dice (mean ± std)	Net overlap (mean ± std)
Training (n=1390)	0.43±0.27	0.42±0.26
Testing (n=459)	0.45±0.26	0.44±0.25
STIR 1 (n=140)	0.23±0.22	0.18±0.20
STIR 2 (n=140)	0.48±0.28	0.39±0.26

Supplementary Table 3. Dice and Net Overlap of the top parameters′ configuration of the modified c-fussy method over all datasets.

SUPPLEMENTARY FIGURES

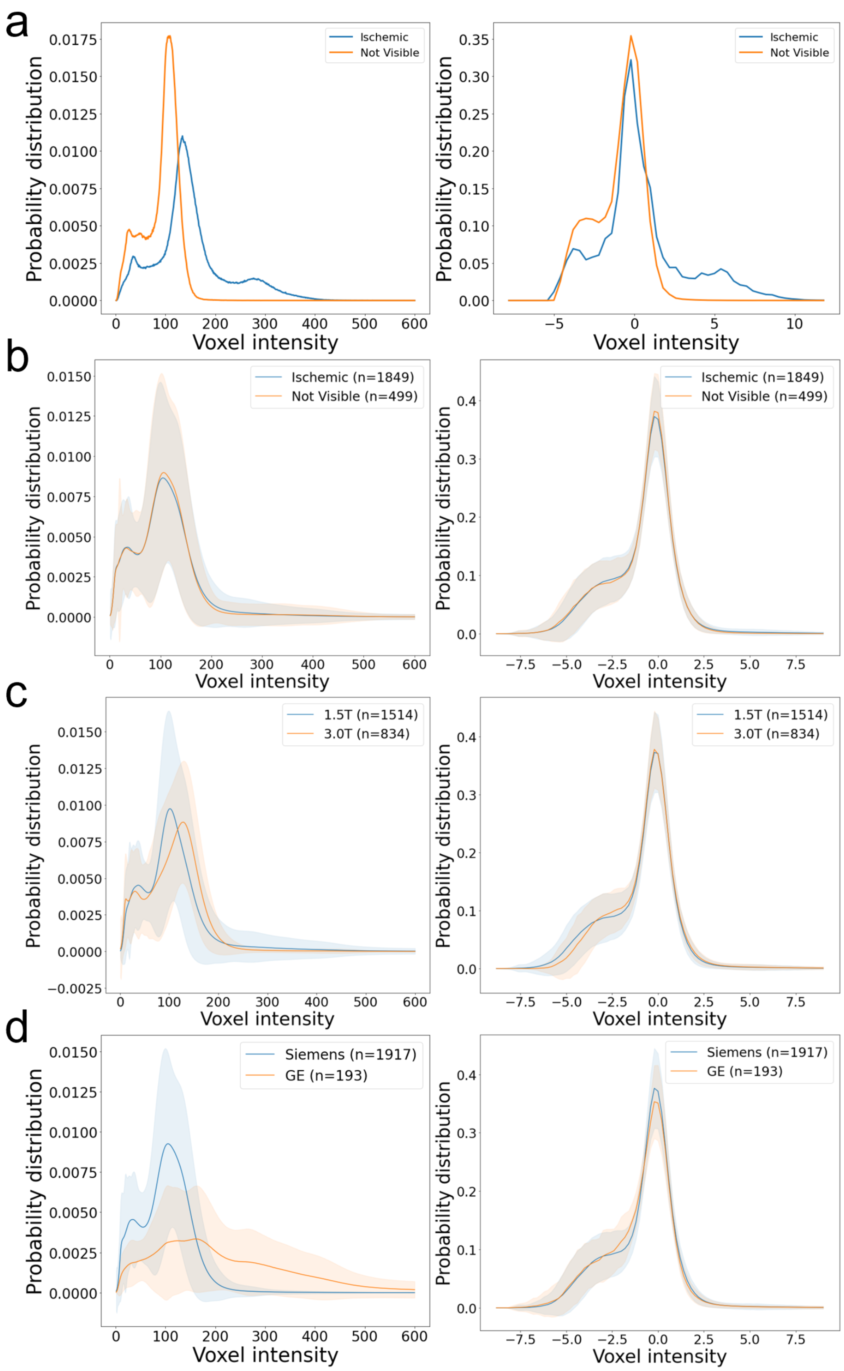

Supplementary Figure 1. Title: Probability distribution of DWIs' voxel intensity prior and post intensity normalization. Legend: Probability distribution (y axis) of DWIs' voxel intensity (x axis) prior-to (first column) and post-to (second column) intensity normalization. Figure (a) shows the distributions of DWI intensities of a selected sample with ischemic lesion (blue) and one with "not visible" lesion (orange). Figure (b), (c), and (d) show the distributions of DWI intensities in groups according to presence of visible ischemic abnormality (b), magnetic fields (c), or scanner manufacturers (d). The solid line is the average group distribution, the shadowed area is within 1 standard deviation from average.

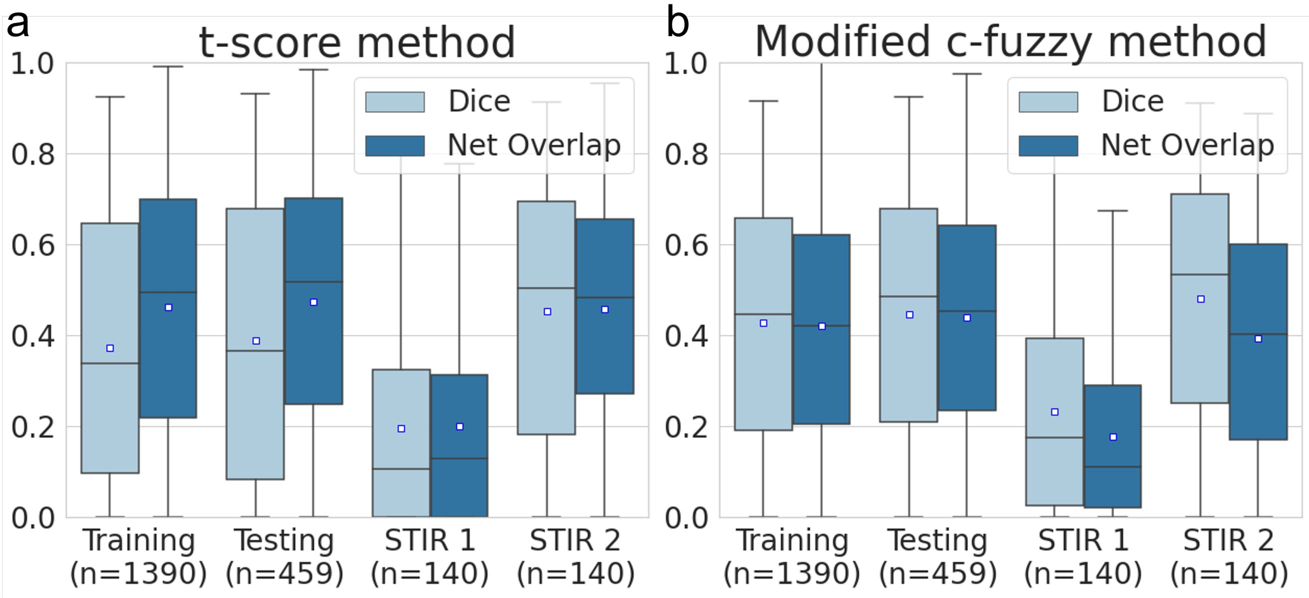

Supplementary Figure 2. Title: The performance of classic models. Legend: The boxplots of Dice and Net overlap for (a) the classic t-score method and (b) the modified c-fuzzy method in Training, Testing, STIR 1 and STIR 2 datasets. In each Whisker's boxplot, the white square indicates the average, the black horizontal line indicates the median. The whisker is a representation of a multiple (1.5) of interquartile range (IQR).

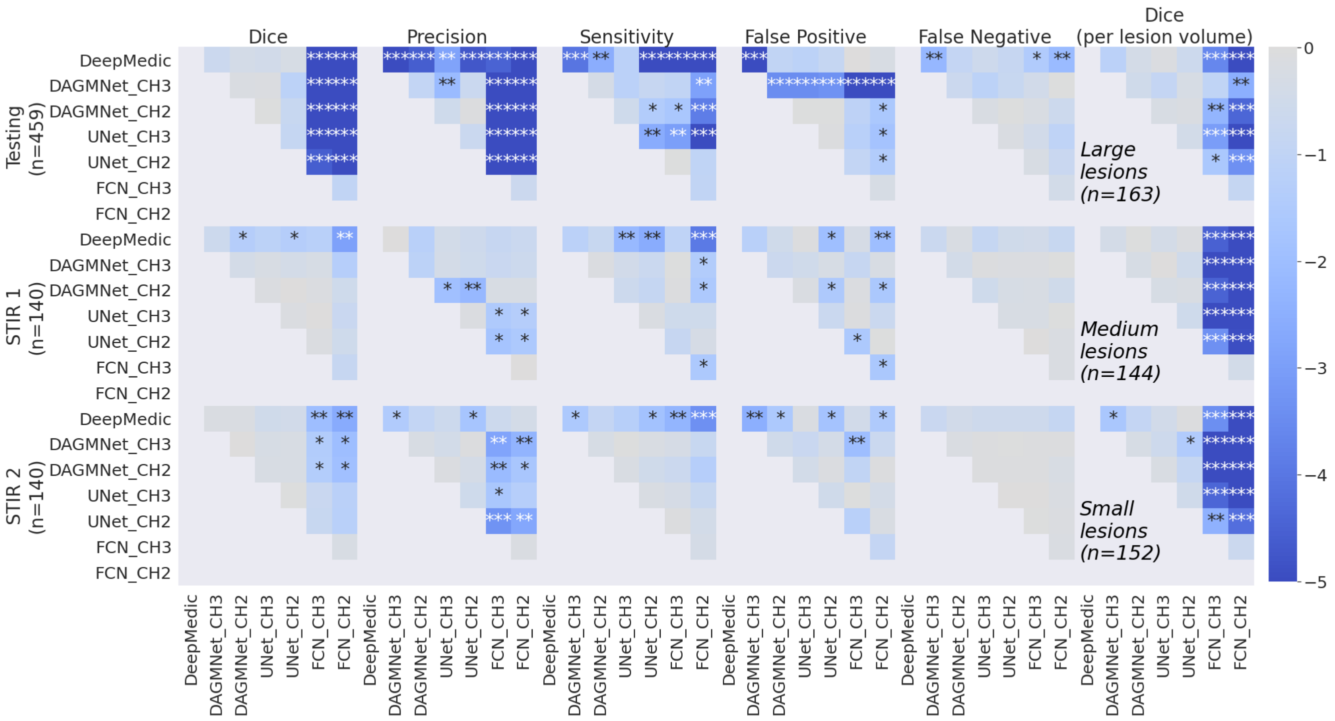

Supplementary Figure 3. Title: Statistical difference between model's performance. Legend: The heapmap of Anova p-values between models' performance in the Testing, STIR 1, and STIR 2 datasets. The rightest column is for the models Dice in the Testing dataset, stratified by lesion volume. $*: p < 0.05$, $**: p < 0.01$, $***: p < 0.001$. The colorbar is in the scale of 10^x, for $x \in [0, -5]$.

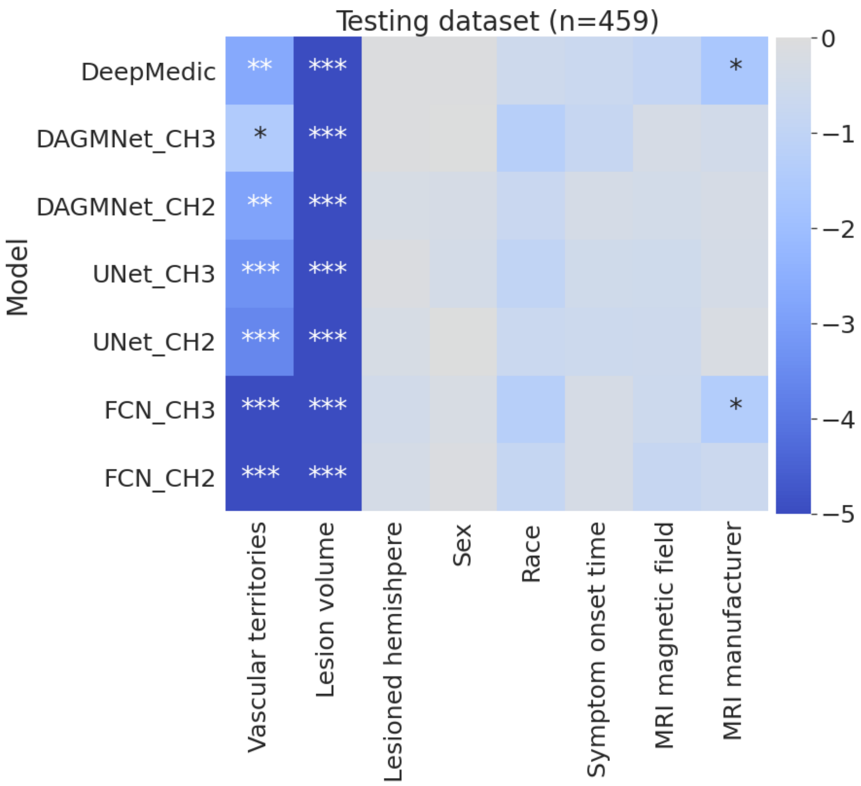

Supplementary Figure 4. Title: Statistical difference in models' performance over the Testing dataset (n=459) according to data profiles. Legend: The heatmap of the Anova p-values for each model, over different profiles. $* : p < 0.05$, $** : p < 0.01$, $*** : p < 0.001$. The colorbar is in the scale of 10^x, for $x \in [0, -5]$.
