# Supplementary material for: Deep learning-based detection and segmentation of diffusion abnormalities in acute ischemic stroke
Source: Commun Med (Lond). 2021 Dec 16;1:61. doi: 10.1038/s43856-021-00062-8 (PMC9053217; doi:10.1038/s43856-021-00062-8)
Supplement: Supplementary file 3 — Reporting Summary [file 43856_2021_62_MOESM3_ESM.pdf]

nature research

Corresponding author(s): Andreia V Faria

Last updated by author(s): Aug 30, 2021

Reporting Summary

Nature Research wishes to improve the reproducibility of the work that we publish. This form provides structure for consistency and transparency in reporting. For further information on Nature Research policies, see our Editorial Policies and the Editorial Policy Checklist.

Statistics

For all statistical analyses, confirm that the following items are present in the figure legend, table legend, main text, or Methods section.

n/a	Confirmed	
☐	☒	The exact sample size (*n*) for each experimental group/condition, given as a discrete number and unit of measurement
☒	☐	A statement on whether measurements were taken from distinct samples or whether the same sample was measured repeatedly
☒	☐	The statistical test(s) used AND whether they are one- or two-sided<br>*Only common tests should be described solely by name; describe more complex techniques in the Methods section.*
☒	☐	A description of all covariates tested
☒	☐	A description of any assumptions or corrections, such as tests of normality and adjustment for multiple comparisons
☐	☒	A full description of the statistical parameters including central tendency (e.g. means) or other basic estimates (e.g. regression coefficient) AND variation (e.g. standard deviation) or associated estimates of uncertainty (e.g. confidence intervals)
☒	☐	For null hypothesis testing, the test statistic (e.g. *F*, *t*, *r*) with confidence intervals, effect sizes, degrees of freedom and *P* value noted<br>*Give P values as exact values whenever suitable.*
☒	☐	For Bayesian analysis, information on the choice of priors and Markov chain Monte Carlo settings
☐	☒	For hierarchical and complex designs, identification of the appropriate level for tests and full reporting of outcomes
☒	☐	Estimates of effect sizes (e.g. Cohen's *d*, Pearson's *r*), indicating how they were calculated

Our web collection on statistics for biologists contains articles on many of the points above.

Software and code

Policy information about availability of computer code

Data collection	n/a. This study was based in retrospective hospital scans, no data was prospectively and specifically collected for this study
Data analysis	All the evaluated methods and models were built with TensorFlow (tensorflow-gpu version is 2.0.0) and Keras (2.3.1) framework on Python 3.6.

For manuscripts utilizing custom algorithms or software that are central to the research but not yet described in published literature, software must be made available to editors and reviewers. We strongly encourage code deposition in a community repository (e.g. GitHub). See the Nature Research guidelines for submitting code & software for further information.

Data

Policy information about availability of data

All manuscripts must include a data availability statement. This statement should provide the following information, where applicable:

- Accession codes, unique identifiers, or web links for publicly available datasets
- A list of figures that have associated raw data
- A description of any restrictions on data availability

The source data for the main result figures in the manuscript are available at https://zenodo.org (DOI 10.5281/zenodo.5579390). The original images used for training and testing the models derive from retrospective clinical MRIs and are not publicly available now, due to their sensitive information that could compromise research participant privacy. These data can be requested with appropriate ethical approval by contacting Dr. Andreia V. Faria (afaria1@jhmi.edu). The STIR data were used under approval from the STIR steering committee for the current study, and so are not publicly available. These data are however available from the STIR / Vista Investigators upon reasonable request to Dr. Marie Luby (lubym@ninds.nih.gov).

Field-specific reporting

Please select the one below that is the best fit for your research. If you are not sure, read the appropriate sections before making your selection.

☒ Life sciences ☐ Behavioural & social sciences ☐ Ecological, evolutionary & environmental sciences

For a reference copy of the document with all sections, see nature.com/documents/nr-reporting-summary-flat.pdf

Life sciences study design

All studies must disclose on these points even when the disclosure is negative.

Sample size	2,348 clinical diffusion weighted MRIs of patients with acute and sub-acute ischemic strokes
Data exclusions	From a total of 2,888 MRIs, 540 were excluded after the quality-control for image analysis or because they showed signals of hemorrhage, resulting in the final sample of 2,348
Replication	The testing set (n=459) was unseen in the training and validation phases. A second external dataset, STIR (n=280), was used to test the generalization of our models in a completely unrelated population
Randomization	The DWIs with ischemic lesions were randomly split into training dataset (n=1,390), which was used to train and validate all subsequent models, and testing dataset (n=459), which was used exclusively for testing models. The lesion profile (e.g., volume, intensity, location) was balanced between these sets, as shown in the manuscript (Table 1).
Blinding	The investigators (and the system) were blind to the "ground-true" lesion mask of the testing sets.

Reporting for specific materials, systems and methods

We require information from authors about some types of materials, experimental systems and methods used in many studies. Here, indicate whether each material, system or method listed is relevant to your study. If you are not sure if a list item applies to your research, read the appropriate section before selecting a response.

Materials & experimental systems

n/a	Involved in the study
☒	☐ Antibodies
☒	☐ Eukaryotic cell lines
☒	☐ Palaeontology and archaeology
☒	☐ Animals and other organisms
☐	☒ Human research participants
☒	☐ Clinical data
☒	☐ Dual use research of concern

Methods

n/a	Involved in the study
☒	☐ ChIP-seq
☒	☐ Flow cytometry
☐	☒ MRI-based neuroimaging

Human research participants

Policy information about studies involving human research participants

Population characteristics	This study included MRIs of patients admitted to the Comprehensive Stroke Center at Johns Hopkins Hospital with the clinical diagnosis of ischemic stroke, between 2009 and 2019. It utilizes data from a dataset,created under waiver of informed consent (IRB00228775). We have complied with all relevant ethical regulations and the guidelines of the Johns Hopkins Institutional Review Board, that approved the present study (IRB00290649). The population reflects the national population of patients with stroke, as shown in our table 2. It includes only adults (>18 years-old). There is slightly preponderance of males (~52%) over females, and african-american/ blacks (~47%) over other races, reflecting our regional population.
Recruitment	This was a retrospective analysis based on the records of the Comprehensive Stroke Center; there was no recruitment.
Ethics oversight	Johns Hopkins Institutional Review Board

Note that full information on the approval of the study protocol must also be provided in the manuscript.

Magnetic resonance imaging

Experimental design

Design type	lesion detection and segmentation in diffusion weighted images. This is not a functional MRI study

Design specifications	n/a
Behavioral performance measures	n/a

Acquisition

Imaging type(s)	diffusion weighted images, DWI
Field strength	1.5 and 3T, four scanner manufacturers, more than hundred different protocols
Sequence & imaging parameters	Our data includes very heterogeneous protocols, acquired over more than 10 years. The use of heterogeneous dataset is part of our premise for robust performance
Area of acquisition	whole brain scan

Diffusion MRI ☒ Used ☐ Not used

Parameters	clinical DWI, b-values 700-1,000

Preprocessing

Preprocessing software	described in methods
Normalization	described in methods
Normalization template	described in methods (MNI)
Noise and artifact removal	n/a
Volume censoring	n/a

Statistical modeling & inference

Model type and settings	described in the manuscript - deep learning nets for lesion detection and segmentaiton
Effect(s) tested	n/a

Specify type of analysis: ☒ Whole brain ☐ ROI-based ☐ Both

Statistic type for inference (See Eklund et al. 2016)	n/a
Correction	n/a

Models & analysis

n/a	Involved in the study
☒	☐ Functional and/or effective connectivity
☒	☐ Graph analysis
☒	☐ Multivariate modeling or predictive analysis
